# Effect of recombinant protein production and release on microalgal fitness and the impact of environmental conditions for localized therapeutic delivery

**DOI:** 10.1186/s13036-025-00525-9

**Published:** 2025-06-04

**Authors:** Felipe Carvajal, Valentina Vargas-Torres, Daniela Becerra, Nicolás González-Quezada, José Tomás Egaña

**Affiliations:** Institute for Biological and Medical Engineering, Pontificia Universidad Católica de Chile, Vicuña Mackenna 4860, Santiago, 7820436 Chile

**Keywords:** Genetically engineered microalgae, Recombinant protein production, Therapeutic oxygenation, Microalgal fitness, Localized protein delivery, *Chlamydomonas reinhardtii*

## Abstract

**Backgroud:**

Genetically engineered photosynthetic microorganisms have been proposed as a therapeutic approach for the localized delivery of oxygen and recombinant proteins to tissues in various pathological conditions. However, the effect of recombinant protein production and secretion on microalgal fitness, as well as the impact of key environmental conditions on their potential therapeutic performance, has not yet been described. Therefore, in this study, the microalga *Chlamydomonas reinhardtii* was genetically engineered to produce and release the reporter protein mVenus and was then challenged by exposure to different media, temperatures, and substrates.

**Results:**

The genetically modified microalgae were able to produce and release the mVenus protein under standard culture conditions without affecting overall fitness, including cell size and shape, growth potential, and oxygen metabolism, compared to the wild-type strain. Under mammalian cell culture conditions, the strains continued to produce and secrete mVenus protein for up to four days at 22 °C, 30 °C, and 37 °C. Additionally, photosynthetic biomaterials containing the engineered microalgae showed continuous recombinant protein release at 30 °C and 37 °C for up to four days.

**Conclusion:**

The microalga *Chlamydomonas reinhardtii* can be genetically engineered to produce and release recombinant proteins without detrimental effects on its fitness, showing therapeutic potential under mammalian culture conditions and within biomaterials designed to promote tissue regeneration. Overall, these findings support the use of genetically engineered photosynthetic microalgae for the localized and controlled release of oxygen and recombinant proteins for several therapeutic applications.

## Introduction

Several chronic conditions require frequent administration of protein-based therapeutics, such as insulin for diabetes [1], coagulation factors for hemophilia [2], and erythropoietin for chronic kidney disease [3]. These treatments often rely on repeated injections or continuous infusion systems, presenting problems such as patient discomfort, risk of infections, fluctuating drug levels, and dependency on healthcare facilities. Additionally, the high costs associated with manufacturing, storage, and distribution of recombinant protein drugs further burden both patients and healthcare systems [4, 5].

A major limitation of current peptide-based treatments is protein instability and rapid clearance from circulation, requiring multiple daily administrations to maintain therapeutic effects [6, 7]. For instance, insulin-dependent diabetes requires precise dosing and timing to regulate blood glucose levels. Yet, even advanced delivery methods like insulin pumps and continuous monitoring devices still demand constant patient vigilance and intervention [8]. Similarly, hemophilia patients must undergo frequent intravenous injections of coagulation factors, often leading to challenges with venous access and long-term adherence to therapy [9]. These challenges highlight the urgent need for alternative self-sustained and long-lasting protein delivery systems that minimize patient burden while ensuring stable therapeutic effects.

A localized protein delivery system offers several advantages over systemic administration, particularly for chronic conditions [10]. By delivering therapeutic proteins directly to the affected site, lower doses may be required, reducing potential side effects and systemic exposure [11, 12]. Localized delivery also helps overcome drug degradation and clearance issues, ensuring sustained therapeutic levels over extended periods [13, 14]. Moreover, localized protein production eliminates the need for repeated dosing, decreasing reliance on patient compliance, which is particularly beneficial for children, young adults, and elderly populations who may struggle with complex treatment regimens [15, 16].

The conventional cellular protein expression systems include bacteria, yeast, and mammalian cells, each offering unique advantages and limitations for localized and sustained protein delivery [17]. Bacterial systems like *Escherichia coli* are widely used due to their rapid growth, cost-effectiveness, and ease of genetic manipulation [18]. However, their inability to perform post-translational modifications (e.g., glycosylation) and the risk of endotoxin contamination limit their suitability for complex therapeutic proteins, necessitating extensive purification [19]. Yeast systems, like *Saccharomyces cerevisiae*, as an eukaryotic system offers advantages like protein folding and glycosylation, making them better suited for the production of biologics such as vaccines and monoclonal antibodies [20, 21]. However, hyperglycosylation can lead to immunogenicity issues [22], and fermentation costs for yeast-based systems remain higher than production in bacterial systems [23]. On the other hand, mammalian cell cultures, such as Chinese hamster ovary (CHO) cells, have also been explored for producing fully functional therapeutic proteins, including monoclonal antibodies and hormones [17, 24], due to their ability to perform complex post-translational modifications [25]. Despite their advantages, mammalian systems are expensive to manage, slow-growing, immunogenic, and require complex culture conditions, making them impractical for implantable or self-sustained protein production. Additionally, batch-to-batch variability and contamination risks pose significant challenges for long-term applications [26].

Beyond conventional microbial and mammalian platforms, alternative eukaryotic systems such as insect cells, plants, and plant suspension cultures have been explored for recombinant protein production. Insect cell systems, particularly those based on baculovirus expression vectors, offer safety, scalability, and flexible design. However, their glycosylation patterns differ from those of higher eukaryotes, potentially affecting therapeutic efficacy. Although recent glycoengineering efforts have improved this limitation, these strategies often increase production cost and complexity [27, 28]. Plants present a safe and scalable platform, but they generally display lower productivity than microbial or mammalian systems [29]. Moreover, their longer growth cycles and dependency on specific environmental conditions can limit their use for rapid or large-scale protein production [30]. In contrast, plant suspension cells combine the advantages of plant-based expression with better control over culture conditions and the ability to perform human-like glycosylation. Nevertheless, they typically exhibit slow growth rates and higher production costs compared to microalgae [31, 32].

Given the limitations of conventional microbial systems, and the technical challenges in using other eukaryotic production platforms, eukaryotic microalgae such as *Chlamydomonas reinhardtii* have arisen as an attractive alternative platform for biotechnology and synthetic biology applications [33]. Unlike bacterial systems, *C. reinhardtii* possesses intracellular compartments and organelles that enable post-translational modifications necessary for functional protein expression [34]. Additionally, these microorganisms are generally recognized as safe (GRAS) by the FDA, making them promising candidates for biomedical applications [35]. Compared to yeast expression systems, *C. reinhardtii* can avoid issues related to hyperglycosylation, which can lead to immunogenicity in therapeutic proteins [36]. In comparison to these systems, including insect [37], plant [38], and mammalian cells [39], *C. reinhardtii* represents a cost-effective and versatile platform. It can grow in minimal media, under phototrophic, mixotrophic, or heterotrophic conditions, and requires simpler infrastructure with shorter production times, making it a promising candidate for scalable, localized therapeutic protein delivery.

Several studies have demonstrated the feasibility of embedding *C. reinhardtii* within various hydrogel formulations, including silica, gelatin methacryloyl, and alginate [40–42]. Due to their water retention capacity, these hydrogels support prolonged photosynthetic activity and oxygen production, creating a localized oxygenation system for hypoxic tissue treatment and enhancing the survival of co-encapsulated mammalian cells in hypoxic environments [41]. Their ability to retain water also enables the immobilization of microalgae alongside embedded molecules, such as growth factors like VEGF [42].

Beyond hydrogels, alternative immobilization strategies have been explored for localized oxygen release, including the incorporation of *Chlamydomonas* into surgical sutures [43], collagen-based dermal scaffolds [44], and electrospun polymer fibers [45]. Genetically engineered microalgae seeded onto commercial surgical sutures exhibited sustained oxygen production and the release of recombinant human growth factors such as VEGF and PDGF-BB under microalgal standard culture conditions [43]​. Additionally, incorporating genetically modified microalgae into a hydrogel dressing resulted in the release of recombinant VEGF in a saline medium [46]. These findings highlight the potential of *Chlamydomonas*-based biomaterials as a platform for controlled oxygen delivery and therapeutic molecule production in biomedical applications. By engineering living cells to synthesize and secrete proteins at the site of action continuously, this approach could eliminate the need for external drug delivery and systemic administration, significantly reducing logistical and financial burdens.

Fluorescent proteins are widely used as trackable markers in synthetic biology to monitor gene expression and protein localization [47, 48]. Among the fluorescent proteins expressed in *C. reinhardtii*, mVenus is particularly suitable due to its high stability, rapid maturation, and ease of detection [49, 50]. Moreover, its absorption and emission wavelengths do not overlap with chlorophyll, minimizing autofluorescence and enabling clear visualization [51].

As a proof-of-concept for localized recombinant protein production, this study engineered *C. reinhardtii* to produce and release mVenus, analyzing its impact on microalgal fitness. Additionally, the effects of different culture conditions mimicking mammalian environments on recombinant protein expression and release were evaluated, aiming to assess the system’s potential as a self-sustained therapeutic platform for continuous protein synthesis and delivery in biomedical applications.

## Methods

### Plasmid construction

Genetic constructs were assembled using the uLoop and the *Chlamydomonas* MoClo toolkit [52]. The uLoop system was employed for iterative DNA assembly, ensuring flexibility in genetic design [53]. Plasmid backbones, promoters, coding sequences, and terminators were combined following standardized overhangs to generate functional expression cassettes. Reaction mixtures were prepared according to the authors [53], utilizing Type IIS restriction enzymes and T4 DNA ligase (NEB, USA). Ligated products were transformed into competent TOP10 *Escherichia coli* via heat shock, and colony PCR and sequencing confirmed successful clones.

Endogenous introns were incorporated into coding sequences to optimize transgene expression in *C. reinhardtii*. Intron-containing constructs were designed based on established intron-mediated enhancement principles, ensuring improved transcriptional and translational activity [54, 55].

For the paromomycin resistance cassette, APHVIII coding sequence was used, transcriptionally controlled by the promoter and terminator from beta-Tubulin (β-TUB) [52, 56]. For the mVenus expression cassette, mVenus coding sequence was used, transcriptionally controlled by nuclear synthetic promoter SAP11 and terminator from RuBisCO small subunit (RBCS2) [52, 57]. The secretion peptide pJP30 was placed in the N-terminal of mVenus in the export plasmid [58]. Backbones used for all cassette insertions were the pCA plasmids previously described [53].

### Microalgae culture conditions

Cell-wall-deficient *C. reinhardtii* (cw15-30-derived UVM4) and transformant strains of microalgae were grown under normal conditions [46]. Briefly, cultures were maintained photomixotrophically at room temperature (22 ± 3 °C) in a solid TAP medium containing 1.5% (w/v) agar or in liquid TAP medium. Growth conditions included continuous exposure to white light (30 µE/m²s) with constant agitation at 180 rpm in an orbital shaker. Cell density was determined by spectrophotometry at 550 nm (OD_550_) [59] and was calculated via linear regression with the following formula (obtained from experimental data):

$$\:million\:of\:cells/mL\:=\:(\:OD550\:+\:0.001311)\:/\:\:0.04180$$

For experiments performed under physiologically relevant conditions, the cell density of liquid cultures in the exponential growth phase was determined, and microalgae were harvested by centrifugation (2000 × g for 10 min). The resulting pellets were resuspended in DMEM mammalian cell culture medium (Thermo Fisher, USA) supplemented with 2.5% (v/v) fetal bovine serum (FBS) (Diagnovum, Germany) at a density of 1 × 10^7^ cells/mL. The cultures were maintained for up to 4 days under continuous white light exposure (30 µE/m²s) with constant agitation at 180 rpm in an orbital shaker under three different temperature conditions (22 °C, 30 °C, and 37 °C).

### Nuclear transformation of *C. reinhardtii* by glass beads

Experiments were performed as previously described [60]. Briefly, the glass bead-mediated transformation of *C. reinhardtii* involves growing cells in TAP medium to mid-log phase (OD_550_ ~ 0.3–0.5), followed by harvesting 1 × 10^8^ cells via centrifugation at 2000 × g for 10 min. The pellet is then resuspended in 300–500 µL of fresh TAP medium, and the transformation mix is prepared by adding 0.5–1 µg of purified plasmid DNA, linearized by XbaI restriction enzyme digestion (Thermofisher, USA), along with approximately 100 mg of sterile 425–600 μm glass beads (Sigma, USA). The mixture was then vortexed at maximum speed for 15 s to facilitate DNA uptake. After vortexing, 6 mL of TAP medium was immediately added to allow cell recovery, and the culture was incubated under standard conditions (22–25 °C, continuous light exposure) for 6 h before plating on selective TAP agar. Transformed colonies typically appear within 7–10 days. As the *Chlamydomonas* strain reported in this paper is cell-wall deficient, no autolysin treatment was necessary to enhance DNA uptake. To confirm successful transformation, colonies were screened via antibiotic resistance, PCR, and fluorescence following protocols described in this section.

### Molecular analysis of *C. reinhardtii* cells

Total genomic DNA was extracted using previously published protocols [61]. Briefly, 1 × 10^7^ cells were resuspended in 5% Chelex-100 resin (Bio-Rad, USA), vortexed for 10 s, and heated at 95 °C for 10 min to lyse cells. Lysates were stabilized in ice and vortexed, and Chelex resin was precipitated by spin down. Total genomic DNA was recovered from supernatant and used for further analysis.

For detection of nuclear transcripts, total RNA was extracted from 1 × 10^7^ cells using TRIZol reagent (Ambion, USA), following manufacturer instructions. Total RNA was treated with DNAseI (Thermofisher, USA) for removal of residual genomic DNA. Reverse transcription reactions were prepared using Oligo (dT) 12–18 primer (Invitrogen, USA) as primer, to ensure cDNA synthesis from polyadenylated nuclear transcripts exclusively, using the high capacity cDNA reverse transcription kit (Applied biosystems, USA), following manufacturer instructions.

PCR reactions were performed using SapphireAmp^®^ Fast PCR Master Mix (Takara Bio Inc., Japan) in a 10 µL total reaction, with 1 µL of genomic DNA or cDNA as template. Primers used for PCR are shown in Table 1.

**Table 1:** DNA primers used for detection on genomic DNA or cDNA of endogenous or Recombinant genes

Primer name	Primer sequence (5’ − 3’)	Expected target size	Reference
Venus_F	GCGCACCATCTTCTTCAAGGAC	497 base pairs from gDNA 168 base pairs from cDNA	This work
Venus_R	TACACGTTGTGGCTGTTGTAGTT		
18S_F	ACCTGGTTGATCCTGCCAG	1800 base pairs	[62]
18S_R	TGATCCTTCYGCAGGTTCAC		
qUBC8_F	GTACAGCGGCGGCTAGAGGCAC	162 base pairs	[63]
qUBC8_R	AGCGTCAGCGGCGGTTGCAGGTATCT		

### Absorbance and fluorescence measurements

Absorbance and fluorescence measurements were performed using a Synergy HTX microplate reader controlled by Gen5 v2.07 software (Agilent BioTek, USA). Whole microalgae cultures were transferred into 96-well microplates, with 200 µL of each sample loaded in duplicate (technical replicates). The microplate was placed in the reader at room temperature. Absorbance was measured at 550 nm and 680 nm to determine cell density and chlorophyll content over time, respectively. mVenus fluorescence was recorded using excitation and emission filters of 500/27 and 540/25, with bottom reading and a gain setting of 80. Results were obtained as relative fluorescence units (RFU). Blank wells containing only the growth medium (either TAP or DMEM supplemented with 2.5% FBS) were included for background correction. In liquid *C. reinhardtii* experiments, fluorescent protein accumulation over time was quantified by measuring whole culture fluorescence. In experiments with biomaterials, mVenus production was measured daily from the supernatant of the biomaterials, after clearing by centrifugation (2000 g for 10 min).

### Oxygen consumption and production measurement

Oxygen consumption and production were measured for all strains of microalgae in TAP medium (1 × 10^7^ cells/mL, 22 °C), and physiologically relevant conditions. Thus, microalgae incubated in DMEM-2.5% FBS medium at their respective temperatures were periodically measured for oxygen metabolism using an Oxygraph + System (Hansatech Instruments, UK) at 22 °C, 30 °C, or 37 °C. For this procedure, samples (1 mL) were introduced into the electrode chamber and subjected to 5 min of darkness, followed by 5 min of red (630 nm, 422 µE/m^2^s) illumination. The oxygen consumption and production rates were calculated using the slope of the oxygen concentration curves over time, utilizing a Python script.

### Morphology assessment by bright-field imaging and analysis

Microalgae suspensions were prepared in TAP medium at 1 × 10^7^ cells/mL and then analyzed by optical microscopy. For imaging, 5 µL of each sample was placed on a glass slide, and bright-field images were acquired using a ZEISS Primovert optical microscope (ZEISS, Germany) equipped with a 40× objective and a standard digital camera (MS60, Mshot, China). Feret’s diameter, MinFeret, and circularity measurements of individual cells (at least 400 cells for each strain) were quantified using Fiji Software v2.9.0 [64].

### Flow cytometry

Chlorophyll and recombinant mVenus protein fluorescence were measured by flow cytometry. Here, unfixed samples (1 mL) were periodically taken from cultures and kept at 4 °C for up to 3 days until analysis. A cell death control was included by heating *C. reinhardtii* at 85 °C for 10 min, to determine the baseline level of fluorescence, and setting the gate for each probe for live cell populations (chlorophyll and mVenus fluorescent protein). Samples were acquired at 1 × 10^5^ events per sample (BD FACSCanto II Analyzer, Becton Dickinson, USA). The obtained data was analyzed using FlowJo v10 software (BD Life Sciences, USA). For each experiment, the mVenus gate was set based on a negative control (WT strain cultured in TAP medium), ensuring that background mVenus-positive cells did not exceed 0.05%.

### Photosynthetic biomaterials

Surgical sutures seeded with microalgae were prepared as described previously [43]. Briefly, Vicryl Polyglactin 910 suture threads (Ethicon, USA) 3.5 (European Pharmacopoeia, equivalent to 0.35 mm) were used. The threads were cut into 3 cm segments under sterile conditions. These segments were uncoiled and immersed in a microalgae suspension at a concentration of 5 × 10⁷ cells/mL, then incubated at room temperature under continuous illumination (standard culture conditions) without agitation. After 24 h, the *C. reinhardtii*-coated thread pieces were transferred to a 24-multiwell plate containing fresh TAP medium and maintained in culture for 7 days. Macroscopic images were obtained in a stereoscope (Leica S6D, Germany) using a conventional digital camera (MS60, Mshot).

Alginate-based wound dressings containing microalgae were prepared as previously reported [46]. In summary, sodium alginate solution was transferred into a sterile tube, followed by the addition of calcium carbonate. Then, TAP containing microalgae at a concentration of 5 × 10⁷ cells/mL was added to obtain 1 × 10⁷ cells/cm^2^ for a 2 mm thick hydrogel. Polymerization was initiated by incorporating D-(+)-Gluconic acid δ-lactone (GDL) (Sigma, Germany) solution into the mixture. 2 mL of this mixture was then incorporated in a single well of a 6-well culture plate containing a single-layer of commercially available woven cotton sterile gauze (Reutter, Chile). After allowing the gel to set overnight, the hydrogels were immersed in a 2% calcium chloride solution (Sigma, Germany) for 10 min, washed with TAP solution, and 6 mm diameter punches were obtained for further analysis and experiments. Fabricated hydrogels were moved to 24-wells cell culture plates and incubated with 500 µL of DMEM- 2.5% FBS at the corresponding temperature. Supernatants were collected every 24 h and directly measured using a Synergy HTX microplate reader as described previously.

Collagen-based dermal scaffolds were prepared following previously established protocols with minor modifications [44]. Specifically 6 mm diameter scaffolds (Integra dermal regeneration template, Integra LifeSciences, USA) were slightly dried on a sterile gauze and placed silicone face-down in sterile culture 24-well plates. Microalgal cells were seeded at a density of 5 × 10^6^ cells/cm^2^ in the scaffolds and mixed in a 1:1 ratio with human fibrinogen (EVICEL, Johnson & Johnson, USA). Next, human thrombin (EVICEL, Johnson & Johnson, USA) was added to the scaffolds. The resulting scaffolds were left undisturbed for 30 min to ensure complete polymerization of the fibrin, before being submerged in 500 µL of sterile TAP medium. Scaffolds were then maintained under constant white light exposure at room temperature (22 ± 3 °C) for 4 days before further experiments. Fabricated scaffolds were moved to 24-wells cell culture plates, and incubated with 500 µL of DMEM- 2.5% FBS at the corresponding temperature. Whole supernatants were collected every 24 h, centrifuged at 2000 × g for 10 min, and measured using a Synergy HTX microplate reader as described previously.

### Fluorescence imaging and analysis

Liquid samples (1 × 10^7^ cells/mL in TAP medium) were mounted into round 25 mm microscope cover glasses. Fluorescence images were obtained in a ZEISS confocal laser scanning microscope LSM 880 with Airyscan (ZEISS, Germany) detection using filter sets for excitation/emission 561 nm/595 nm for chlorophyll and excitation/emission 488 nm/516 nm for mVenus. Images were acquired using a 63× oil immersion objective in z-stack mode (18 steps, z-stacking step size: 0.3 μm). Images were processed with Fiji software [64] by deconvolving with the PSF generator and DeconvolutionLab2 plugins (Richardson-Lucy algorithm with 10 iterations), and aligned with the StackReg plugin selecting the Rigid Body transformation. Then, the fluorescence of the green channel was adjusted (decreasing the brightness) to avoid overlapping background signal of the chloroplast.

For dermal scaffolds, samples were mounted on a 12-well plate and fluorescence images were obtained with a Cytation5 cell imaging multi-mode reader and microscope (Agilent BioTek, USA) at 20× in z-stacking imaging modality (18 steps, z-stacking step: 6 μm) employing DAPI (excitation 377/50, emission 447/60), YFP (excitation 500/24, emission 542/27), and Cy5 (excitation 628/40, emission 685/40) filters. For the alginate-based wound dressings, the same protocol was followed, but without considering the DAPI fluorescence. Surgical sutures were mounted on a 6-well plate, and fluorescence images were also acquired in the Cytation5 system in z-stacking imaging modality at 20 × (6 steps, z-stacking step size: 6 μm). Images of biomaterials were processed with Fiji Software [64] by deconvolving with the PSF generator and DeconvolutionLab2 plugins (Richardson-Lucy algorithm, with 10 iterations for scaffolds and sutures, and 20 for the hydrogel dressings), followed by the Z-projection process.

### Statistical analysis

Data represents at least three independent experiments (N). Statistical analyses were executed using GraphPad Prism 8.3 software (GraphPad Software). The statistical assessment involved determining data normality via the Shapiro-Wilk test, followed by Kruskal-Wallis test, One- or Two-way repeated measurements (RM) ANOVA, and Tukey’s multiple comparisons as applicable. Significance between groups was established at *p* < 0.05. Further details are provided in the legend of each figure.

## Results

### mVenus expression in *C. reinhardtii* and its impact on microalgal fitness

**Fig. 1:**
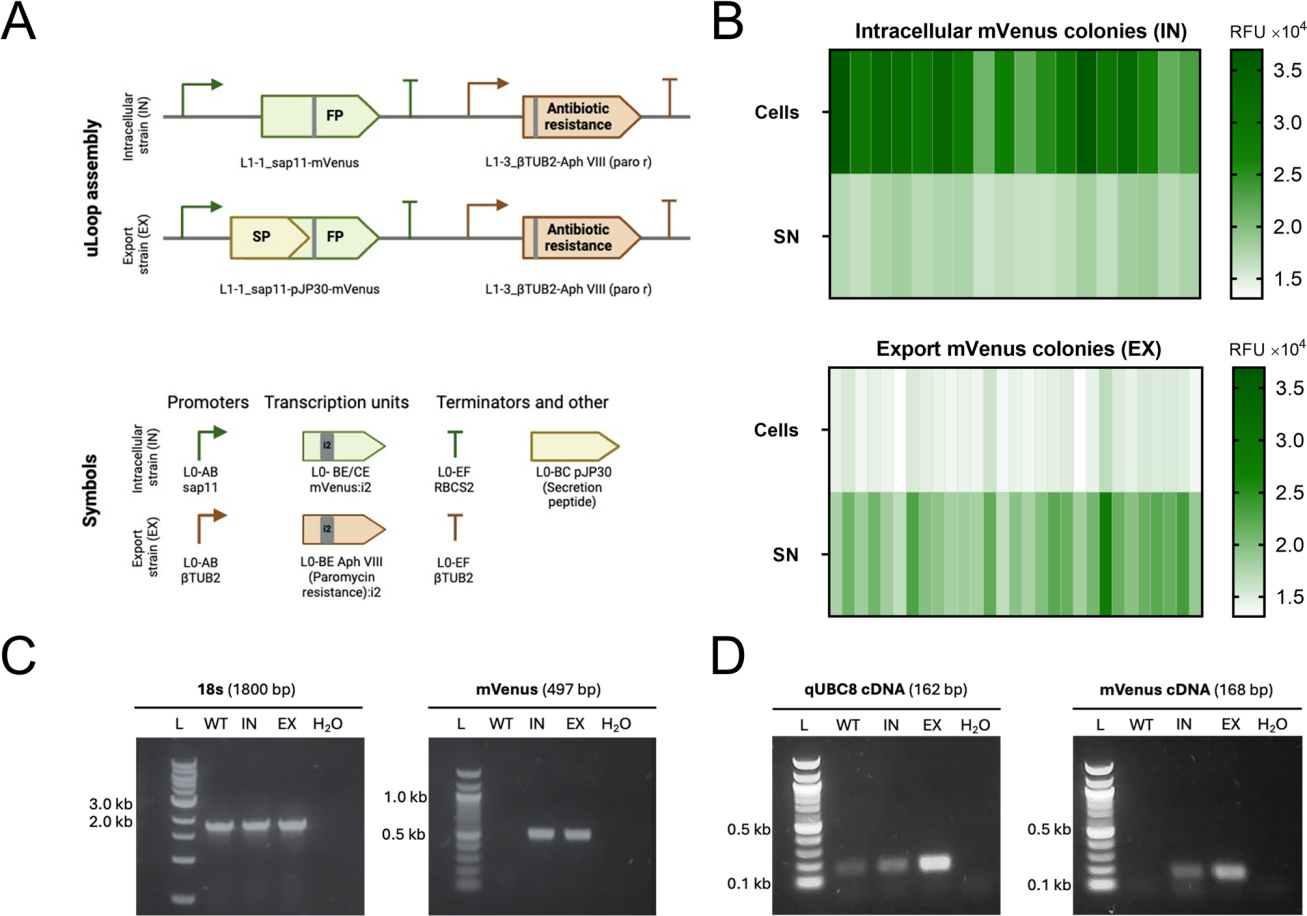
Molecular constructs and expression of recombinant mVenus in *C. reinhardtii.***(A)** Schematic representation of the genetic constructs assembled using uLoop assembly and MoClo genetic parts. **(B)** Fluorescence screening of mVenus-expressing colonies to identify those with highest signal intensity, where each column represents an individual colony (16 and 27 colonies for IN and EX, respectively). Selected colonies for the intracellular (IN) and export (EX) strains, along with a wild-type colony (WT), were analyzed for **(C)** genomic DNA, targeting the ribosomal 18 S subunit of *C. reinhardtii* and mVenus gene, and **(D)** cDNA for qUBC8 and mVenus nuclear transcripts. SN: supernatant; i2: intron 2.

A schematic representation of the molecular design is shown in Fig. 1A, illustrating the stepwise assembly of the genetic elements into functional expression cassettes. Using this approach, two genetically engineered strains were generated: one to accumulate mVenus intracellularly (IN strain) and another to export it (EX strain). In addition, the parental wild-type *C. reinhardtii* UVM4 strain was used as a control (WT strain).

After transformation, 78 and 150 colonies of IN and EX strains, respectively, were picked, and the fluorescence of mVenus was evaluated in both cells and supernatants (Fig. 1B). Fluorescent positive colonies were obtained at rates of 21% and 18% for each strain, respectively. Next, a single colony from each strain exhibiting the strongest fluorescence profile was selected and further characterized by PCR using the primers listed in Table 1. As shown in Fig. 1C and D, bands of the expected sizes were obtained for each amplicon, both from genomic DNA and cDNA of nuclear transcripts.

**Fig. 2:**
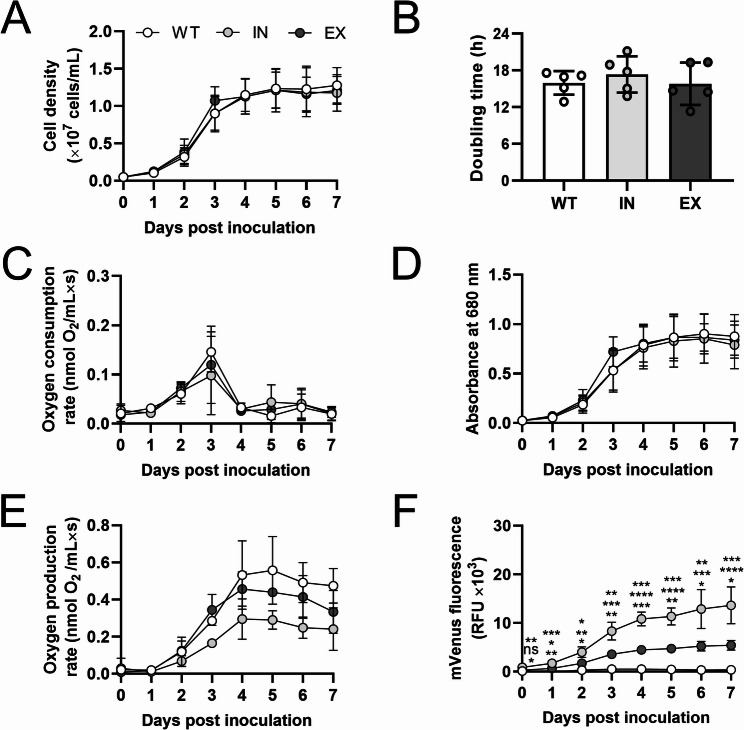
Physiological fitness evaluation of recombinant *C. reinhardtii* strains. **(A)** Growth curves. **(B)** Calculated doubling times (in hours) during the exponential phase modeled using a Malthusian growth approach. **(C)** Oxygen consumption was measured as an indicator of mitochondrial activity. **(D)** Chlorophyll content was quantified as absorbance at 680 nm. **(E)** Oxygen production rates were assessed to evaluate photosynthetic capacity. **(F)** Fluorescence of recombinant mVenus protein over time. Statistical analyses were performed using Two-way RM ANOVA followed by Tukey’s multiple comparison tests in **(A)**, **(C)**, **(D)**, **(E)** and **(F)**, and the Shapiro-Wilk test followed by One-way ANOVA in **(B)**. Data are presented as mean ± SD, with *N* = 5 for **(A)**, **(B)**, **(D)**, and **(F)** and *N* = 3 for **(C)** and (E). Statistical significance is indicated by * *p* < 0.05, ** *p* < 0.01, *** *p* < 0.001, **** *p* < 0.0001 and “ns” or the absence of indicators denotes non-significant differences. In (F), the significance between groups is as follows: WT vs. IN (upper), WT vs. EX (middle), and IN vs. EX (lower), whereas **(A)**, **(B)**, **(C)**, **(D)** and **(E)** showed no significant differences.

Then, experiments were performed to determine whether the expression and secretion of the recombinant mVenus affect the fitness of *C. reinhardtii*. First, the growth dynamics of the strains were measured, showing similar curves for all groups, with well-defined lag, exponential, and stationary stages (Fig. 2A). The fit of the curves to a Malthusian model (prediction of population growth based on the exponential phase) was analyzed at day 3 (approximately 1 × 10^7^ cells/mL for all strains), obtaining non-significant differences in average doubling times with values of 15.95 h (R^2^ = 0.88) for WT, 17.34 h (R^2^ = 0.89) for IN, and 15.80 h (R^2^ = 0.93) for EX strains (Fig. 2B). Next, oxygen consumption was quantified as a direct measure of mitochondrial activity, with no significant differences observed among the strains (Fig. 2C). Interestingly, a peak at day 3 was observed, correlating with the exponential growth phase. To evaluate photosynthetic capacity, chlorophyll content (Fig. 2D) and oxygen production rates (Fig. 2E) were measured over seven days showing no significant differences among strains. Lastly, the total fluorescence of recombinant mVenus was evaluated over time (Fig. 2F), revealing three distinctive fluorescence patterns. The IN strain showed significantly higher fluorescence than the EX strain, whereas the WT strain showed a low and stable background signal. Notably, fluorescence in both engineered strains closely followed the growth pattern, with a mild increase during the first day, a steeper rise over the next two to three days, and a plateau toward the end of the measurement period.

**Fig. 3:**
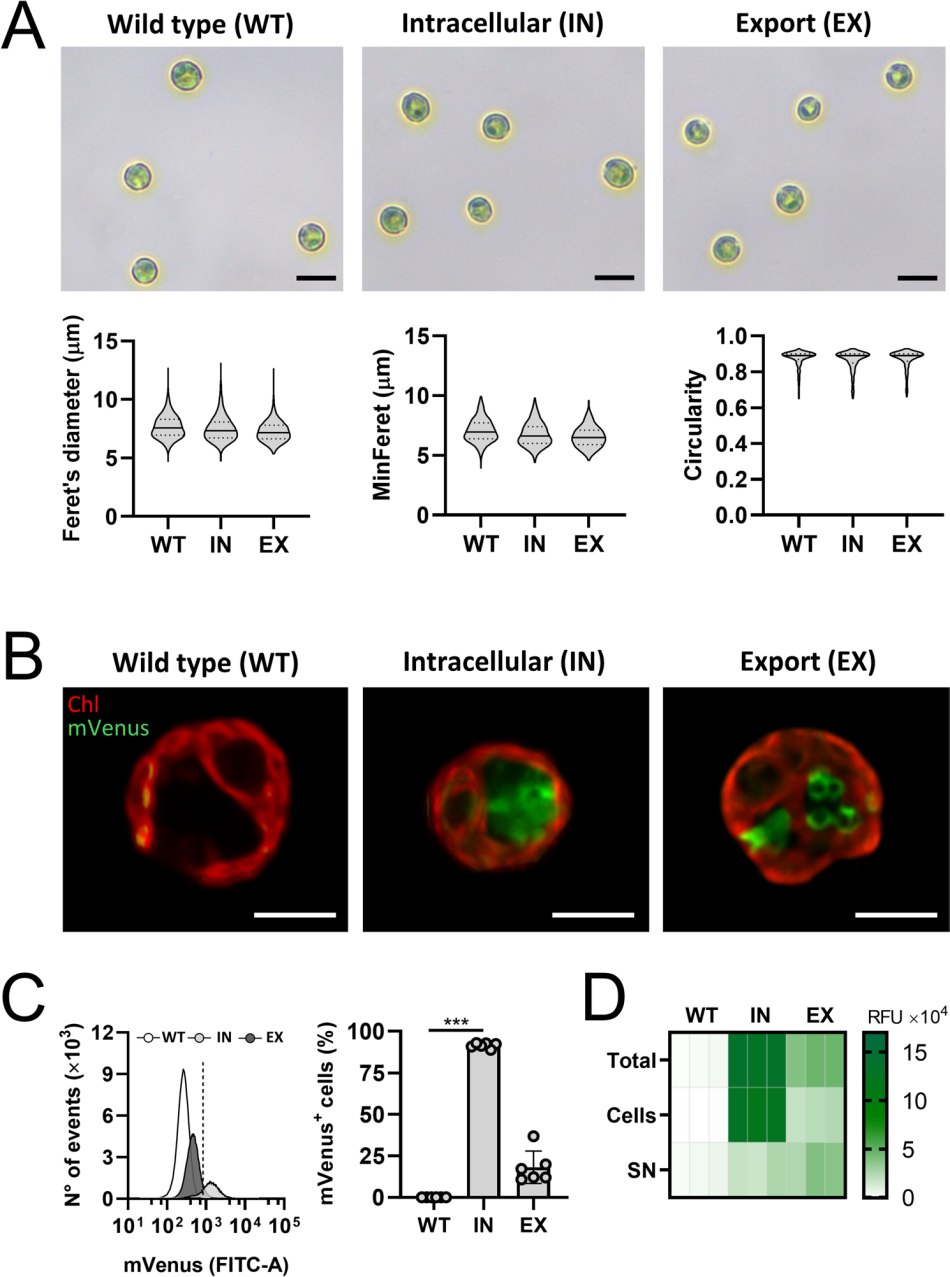
Recombinant mVenus production in standard culture conditions. Microalgae were harvested during the exponential growth phase and resuspended in fresh TAP medium at 1 × 107 cells/mL. **(A)** Representative brightfield images showing microalgal morphology and quantification of particle length (Feret’s diameter), width (MinFeret), and circularity. In the violin plots, the solid line represents the median, while the dotted lines indicate the interquartile range, based on the analysis of at least 400 cells per strain (*N* = 3). **(B)** Confocal fluorescence images of the strains show the intracellular distribution of chlorophyll (Chl, red) and mVenus (green). **(C)** Representative histogram of mVenus fluorescence (left panel), where the dotted line indicates the threshold used to identify mVenus-positive cells, and corresponding quantification of mVenus-positive cells (right panel). **(D)** Total mVenus fluorescence of whole cultures (total), cell pellet (cells), and supernatant (SN) from three independent cultures derived from the same colony. Statistical analysis was performed using the Shapiro-Wilk test and the Kruskal-Wallis test in (**C**, right panel). Data are presented as Mean ± SD with *N* = 6 in (**C**, right panel). Statistical significance in (**C**, right panel) is indicated by *** *p* < 0.001 or the absence of indicators denotes non-significant differences. Scale bars represent 10 μm in **(A)** and 5 μm in **(B)**

After assessing the potential effect of mVenus production on the fitness of recombinant strains, microalgae were cultured in standard conditions (TAP medium at 22 °C with continuous white light exposure) for further characterization. Morphological analysis showed that gene modification did not affect cell length (Feret’s diameter), width (MinFeret), or circularity (Fig. 3A). In particular, no significant differences were observed among WT, IN, and EX microalgae, with length measurements of 7.67 ± 0.25 μm, 7.52 ± 0.11 μm, and 7.27 ± 0.13 μm, respectively and 7.07 ± 0.33 μm, 6.76 ± 0.16 μm, and 6.55 ± 0.18 μm, respectively.

Next, fluorescence measurements were performed to track mVenus production levels across different strains. As shown in Fig. 3B, WT strain did not exhibit intracellular fluorescence at 516 nm, while transformant *C. reinhardtii* cells exhibited strong intracellular mVenus signal as observed by fluorescence microscopy. It is also noticeable that the transformed strains presented differences in the intracellular distribution of the mVenus signal, with a cytoplasmic signal in the IN strain, and the presence of a condensed fluorescent signal in intracellular vesicle-like structures in the EX strain. It is worth noting that in both IN and EX strains, the signal is found only in the cytoplasm, and not inside the chloroplast, highlighting the successful nuclear transformation of the microalgae, as expected from the design of the molecular constructs described before.

To correlate the fluorescence observed by microscopy, flow cytometry analysis was performed. As shown in Fig. 3C (left panel), in the chlorophyll-high cells, which represents over 99% of the population, mVenus fluorescence intensity increased progressively from the WT, to the EX and IN strains. Moreover, quantification revealed that the proportion of mVenus-positive cells was approximately 0.05 ± 0.02% for the WT strain, 91.53 ± 1.52% for the IN strain, and 18.15 ± 9.78% for the EX strain (Fig. 3C, right panel). Finally, mVenus fluorescence was compared in the different cellular fractions. As expected, only background signal was detected in the WT strain, whereas in the IN and EX strains, fluorescence accumulated in the cytoplasm and supernatant, respectively (Fig. 3D).

### Recombinant mVenus expression under physiologically relevant culture conditions

Overall, the results indicate that the genetic modifications do not impair the general fitness of *C. reinhardtii* under standard culture conditions. However, for clinical applications, environmental stressors may influence their performance. Therefore, the next step was to evaluate their growth capacity and oxygen metabolic dynamics under conditions that better mimic the mammalian microenvironment, including mammalian cell culture media and temperatures ranging from 22 °C to 37 °C.

**Fig. 4:**
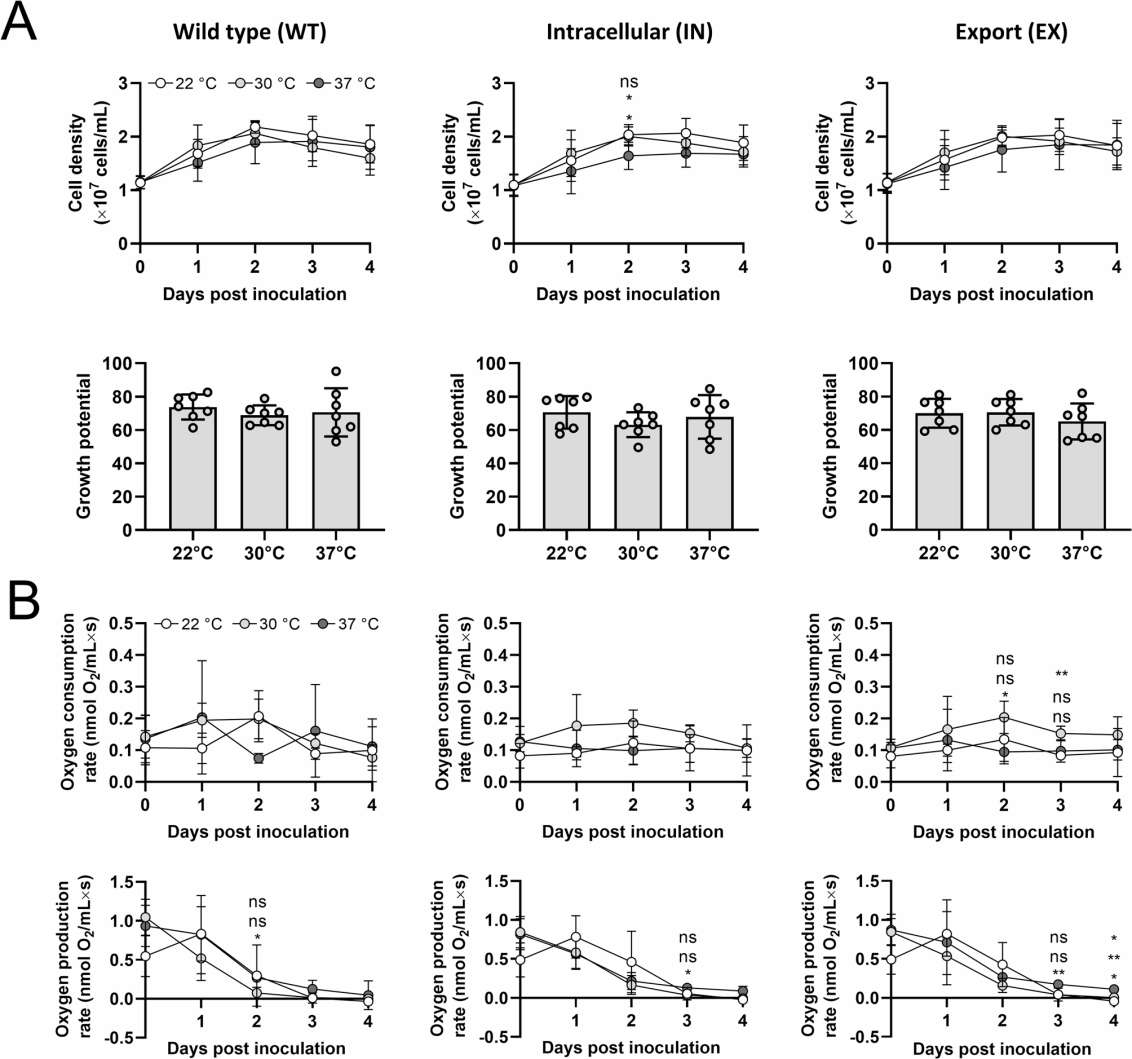
Behavior of recombinant *C. reinhardtii* strains in mammalian cell culture conditions. Microalgal cultures were harvested and adjusted to a density of 1 × 107 cells/mL and then cultured for 4 days at 22 °C, 30–37 °C in DMEM-FBS. **(A)** Growth curves (upper panel) and growth potential (lower panel, measured as the area under the curve). **(B)** Oxygen consumption (upper panel) and production (lower panel) rates. Statistical analysis was performed using Two-way RM ANOVA followed by Tukey’s multiple comparison tests in (**A**, upper panel) and **(B)**, and the Shapiro-Wilk test, and One-way ANOVA test or Kruskal-Wallis test in (**A**, lower panel). Data are presented as Mean ± SD with *N* = 7 in **(A)** and *N* = 4 in **(B)**. Statistical significance is indicated by * *p* < 0.05, ** *p* < 0.01, and “ns” or the absence of indicators denotes non-significant differences. In (**A**, upper panel) and **(B)**, the significance between groups is as follows: WT vs. IN (upper), WT vs. EX (middle), and IN vs. EX (lower)

Results show that all strains display similar growth behavior across all temperatures, reaching a maximum cell density of about 2 × 10^7^ cells/mL at day 2, followed by a slight decline towards day 4 (Fig. 4A, upper panel). The only statistically significant difference was observed in the IN strain on day 2, where growth at 37 °C was reduced compared to 22 °C and 30 °C, a trend reversed in subsequent days. Overall, no significant differences were detected among strains at any temperature, as confirmed by the quantification of their growth potential (area under the curve) under these culture conditions (Fig. 4A, lower panel). Under the same culture conditions, oxygen consumption and production were evaluated as indicators of mitochondrial and chloroplast activity, respectively. Oxygen consumption rates (Fig. 4B, upper panel) showed no significant differences between the parental and modified strains, ranging from 0.1 to 0.2 nmol O_2_/mL×s. However, significant differences for the EX strain were observed at days 2 and 3, where oxygen consumption increased at 30 °C. In contrast, regardless of temperature and strain, the oxygen production rate showed a peak at day 1 and then decays towards day 4, with significant reduction from day 2 for WT, or day 3 for IN and EX strains at 30 °C relative to 22 °C and 37 °C (Fig. 4B, lower panel).

**Fig. 5:**
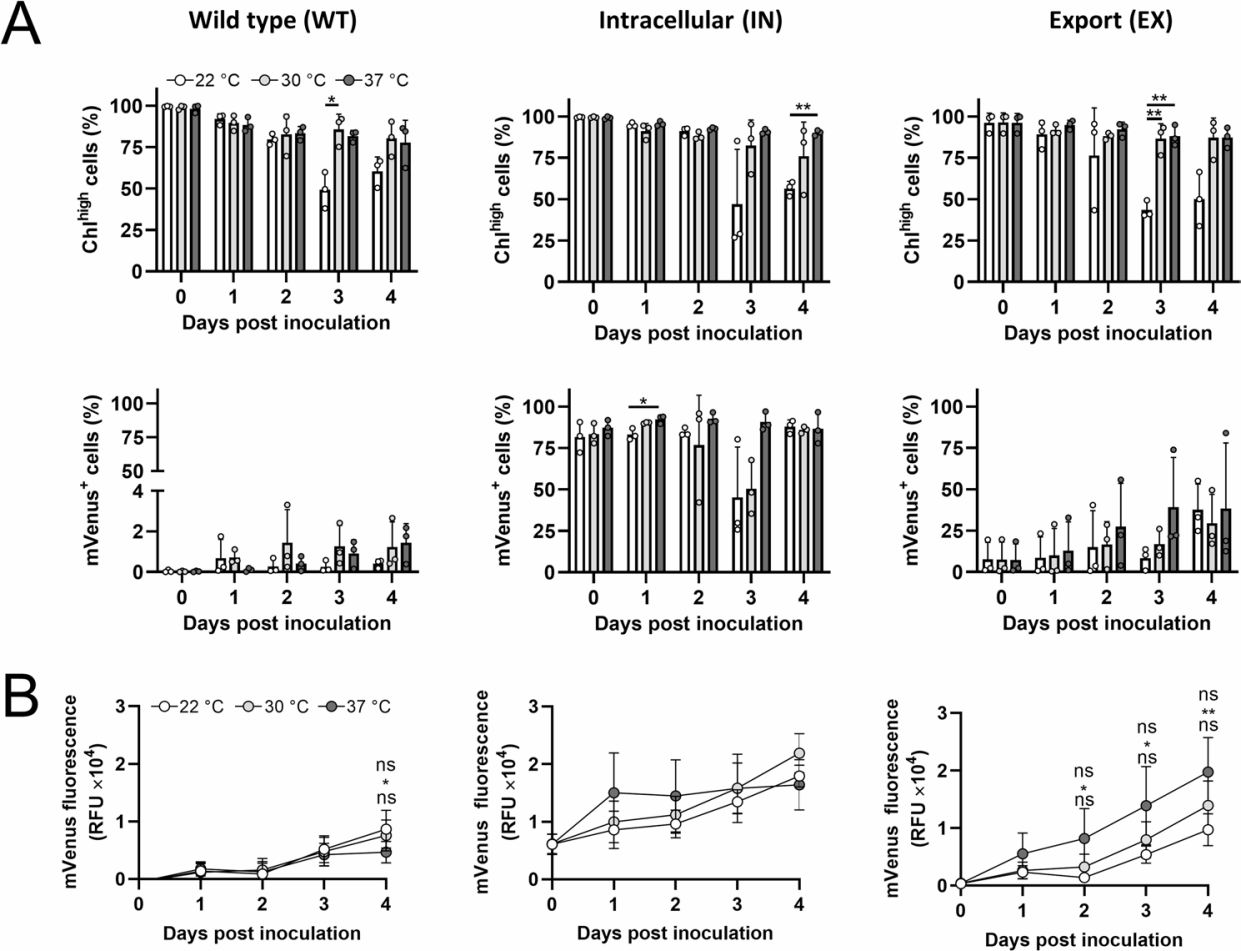
Effect of temperature in recombinant protein production in mammalian cell culture conditions. Microalgal cultures were harvested and adjusted to a density of 1 × 107 cells/mL and then cultured for 4 days at 22 °C, 30–37 °C in DMEM-FBS. **(A)** Flow cytometry analysis showing the percentage of chlorophyll-high cells within the total population (upper panels) and the percentage of mVenus-positive cells (mVenus+) within the chlorophyll-high population (lower panels) over time. **(B)** Fluorescence curves, showing mVenus fluorescence over time. Statistical analysis was performed using a Two-way RM ANOVA followed by Tukey’s multiple comparison tests in **(A)** and **(B)**. Data are presented as Mean ± SD with *N* = 3 in **(A)** and *N* = 7 in **(B)**. Statistical significance is indicated by * *p* < 0.05, ** *p* < 0.01, and “ns” or the absence of indicators denotes non-significant differences. In (**B**, left and right panels) the significance between groups is as follows: 22 °C vs. 30 °C (upper), 22 °C vs. 37 °C (middle), and 30 °C vs. 37 °C (lower).

Afterwards, the effect of temperature and medium composition in mVenus production was evaluated. First, the chlorophyll-high population was quantified by flow cytometry and results suggest that the overall chlorophyll signal was not affected, except for the 22 °C towards day 3 and 4 (Fig. 5A, upper panels). The mVenus signal of the same samples was analyzed by flow cytometry (Fig. 5A, lower panel), showing a remarkable difference among the strains and no differences between temperatures. As expected, WT strain had a background positive population that remained under 4%, while the IN strain denoted the most positive population, remaining over 75% of analyzed events at all timepoints. In comparison, the EX strain had an initial lower mVenus-positive population, which tended to increase over time, representing about 25% of analyzed events on day 4.

When quantifying total mVenus fluorescence over time, results show a small increase in the background signal for the WT strain, while both modified strains exhibit a sustained increase in fluorescent signal up to day 4, denoting the accumulation of the protein (Fig. 5B). When observing the differences in fluorescence at the different temperature conditions between days 1 and 4, a positive variation was observed of 1.18 (22 °C), 1.58 (30 °C), and 1.03 (37 °C) RFU×10^4^ for the IN strain, and 0.93 (22 °C), 1.35 (30 °C) and 1.93 (37 °C) RFU×10^4^ for the EX strain. It is also important to note that the individual curves at each temperature did not show statistical differences in the IN strain, although there was a significant difference in the WT strain between the curves at 22 °C and 37 °C. Notably, the EX strain showed a significant increase in fluorescence from day 2 up to day 4 between the 37 °C and 22 °C conditions (Fig. 5B).

### Biomaterial activation

**Fig. 6:**
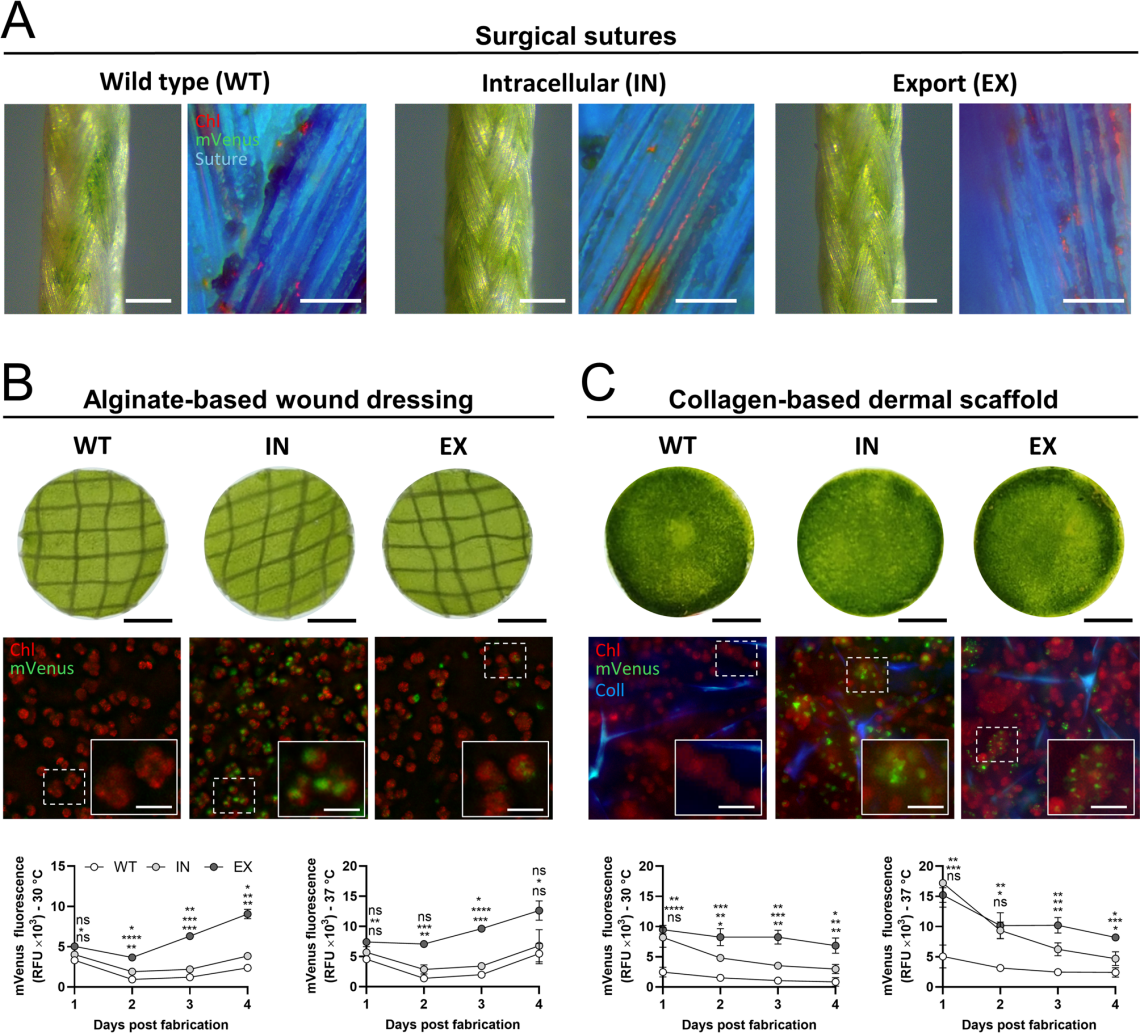
Recombinant protein release from *C. reinhardtii* seeded biomaterials. **(A)** Overview of surgical sutures seeded with microalgae (left panel) and their fluorescence for chlorophyll and mVenus (right panel). **(B)** Hydrogel-based wound dressing. **(C)** Collagen-based dermal scaffold. In **(B)** and **(C)**, from top to bottom: macroscopic view, fluorescence microscopy showing microalgae, and recombinant protein release quantified as mVenus fluorescence in the supernatant. Fluorescent images include a digital zoom at the bottom-right, with the zoom marked by a continuous line and the original image outlined by a dotted line. Statistical analysis was performed using Two-way RM ANOVA followed by Tukey’s multiple comparison tests. Data are presented as Mean ± SD with *N* = 3 for hydrogel dressings and *N* = 4 for scaffolds. Statistical significance is indicated by * *p* < 0.05, ** *p* < 0.01, *** *p* < 0.001, **** *p* < 0.0001 and “ns” or the absence of indicators denotes non-significant differences. In **(B)** and **(C)** the significance between groups is as follows: WT vs. IN (upper), WT vs. EX (middle), and IN vs. EX (lower). Scale bars represent 200 μm in the macroscopic view of sutures, 2 mm in macroscopic images of hydrogel dressings and dermal scaffolds, 50 μm in fluorescence images of sutures and 10 μm in fluorescence images of wound dressing and scaffold (digital zoom). Chl: chlorophyll, Coll: collagen matrix

To add another level of therapeutic complexity, the recombinant strains were incorporated into different previously described photosynthetic biomaterials developed for wound healing [43, 44, 46]. First, their incorporation in surgical sutures was tested, showing a homogeneous distribution of all three microalgae strains and, as expected, a detectable recombinant fluorescent signal for the IN strain (Fig. 6A).

Next, an alginate-based wound dressing and a collagen-based dermal scaffold were seeded, and mVenus release was studied for up to four days at 30 °C and 37 °C. The photosynthetic wound dressings and scaffolds showed an overall green coloration, indicating a homogeneous distribution of the three microalgae strains (Fig. 6B and C, upper panels). Furthermore, fluorescence microscopy images confirm the presence of individual cells with chlorophyll autofluorescence within both biomaterials, while mVenus fluorescence was detected in both recombinant strains (Fig. 6B and C, middle panels).

Finally, supernatants were collected and replaced daily, and the fluorescence at each day was quantified and compared (Fig. 6B and C, lower panels). For the wound dressing, a similar background signal was detected for the WT and IN strain at both temperatures, with no significant differences between days. In contrast, for the EX strain, a significant increase in signal was detected from day 2 to 4, as well as on days 2 and 3 at 30 °C and 37 °C, respectively. Similar results were obtained for the dermal scaffold, where significant differences for the EX strain were observed from day 2 to day 4, and at days 3 and 4 for 30 °C and 37 °C, respectively. Unexpectedly, the IN strain had a significant increase in fluorescent signal from day 1 in both temperatures analyzed, with a progressive reduction in subsequent days.

## Discussion

This study evaluates the potential of *C. reinhardtii* as a platform for recombinant protein production under physiologically relevant conditions. By expressing the mVenus fluorescent protein, the study examines genetic stability, metabolic activity, and protein secretion under various environmental conditions. Additionally, the adaptability of recombinant microalgae to mammalian-like culture conditions was explored, along with their integration into biomaterial-based devices for controlled protein release.

The findings described in this work contribute to the broader goal of developing microalgae-based biofactories for biomedical applications, emphasizing both their adaptability in non-native environments and the modular genetic tools that enable their optimization. Furthermore, this work validates the previously reported high-efficiency synthetic promoter SAP11 [57] and the predicted pJP30-optimized secretion signal [58]. Although alternative high-yield, highly glycosylated secretion signals are available [65], such optimized peptides were not used due to its high glycosylation characteristics, which, despite its high efficiency in secreting proteins from *C. reinhardtii*, can be detrimental in biomedical applications due to potential immunogenicity issues [22, 66].

Fluorescent proteins such as GFP and mVenus have been successfully expressed in different microbial systems, including *E. coli*, *S. cerevisiae*, and the microalgae *C. reinhardtii*. Bacterial systems like *E. coli* are widely used for their high expression levels of fluorescent proteins like GFP [67], but the limitations previously described may affect the production of bioactive proteins. Yeast systems, such as the model *S. cerevisiae*, can also produce fluorescent proteins, with yields of approximately 6 mg/L. Moreover, issues like hyperglycosylation and limited secretion efficiency can occur [68]. In contrast, *C. reinhardtii* combines eukaryotic protein processing capabilities, such as proper folding and disulfide bond formation, with the advantages of microbial scalability and cultivation in a simple culture media. Fluorescent proteins expressed in *C. reinhardtii* have demonstrated sustained expression and stability under a variety of conditions, reaching up to ~ 15 mg/L when optimized with synthetic glycomodules [65]. Moreover, its photosynthetic nature allows it to contribute molecular oxygen, a feature that could offer additional benefits for therapeutic applications in hypoxic environments. Importantly, as mentioned above, *C. reinhardtii* is non-pathogenic and generally recognized as safe, strengthening its appeal for biomedical use in localized delivery of recombinant proteins.

The similar growth curves, chlorophyll absorbance, and oxygen metabolism observed across WT and the engineered strains confirm that no adverse effects arose from the insertion of the DNA fragments into the cellular genome or from recombinant protein production. Furthermore, a conserved metabolic burden was observed across all analyzed strains despite the production of the exogenous protein. The increase in oxygen consumption observed on day three of liquid cultures overlaps with the exponential growth phase, and as the cultures mature, oxygen consumption gradually stabilizes while the cells maintain their ability to produce oxygen under illumination.

Confocal microscopy analysis showed that the intracellular distribution of the mVenus protein differed between engineered *C. reinhardtii* strains. The cytoplasmic localization observed in the IN strain aligns with expectations for non-secreted proteins. In contrast, the vesicle-like structures detected in the EX strain might suggest protein trafficking toward the cellular secretion pathway [58]. Notably, flow cytometry analysis revealed that only about 18% of EX strain cells were gated as mVenus-positive, in contrast to fluorescence microscopy, where most observed cells displayed the characteristic vesicle-like mVenus signal. This observed disparity may arise from the lowered excitation efficacy of the cytometer’s blue laser, which has a wavelength of 488 nm, accounting for only 35% of the maximum mVenus excitation spectrum [69].

Combining efficient protein release and oxygen production capacity might be useful in therapeutic settings to treat hypoxia-related disorders, where the combined use of oxygen and bioactive recombinant peptides can be beneficial, such as for the treatment of chronic wounds, tissue preservation, and tumors [70–73]. By expressing the fluorescent protein mVenus as a proof-of-concept, the system adaptability to conditions that mimic mammalian physiological environments was evaluated, supporting the construction of modular plasmids for gene expression in *C. reinhardtii*, using the uLoop strategy as a rapidly iterative system to explore different regulatory elements.

When exposing any microorganism to challenging environmental conditions, such as different temperatures or modification of its chemical environment, phenomena like delayed growth, detrimental stress responses, or even cell death can occur [74]. While previous studies have examined recombinant protein production in *C. reinhartii* cells under elevated temperatures of about 35 °C [75] to 37 °C [76], the findings of this work go a step further by demonstrating sustained protein production and release as well as metabolic activity in sub-optimal conditions, represented here as mammalian culture conditions. This reveals that the microalga is not only capable of surviving under such challenging conditions but also maintains its metabolic processes, such as photosynthetic oxygen production. The development of recombinant *C. reinhardtii* strains capable of sustained protein production for over four days in non-native conditions represents a promising step toward biomedical applications in hypoxic tissues. Flow cytometry analysis further confirmed population stability, reinforcing the suitability of *C. reinhardtii* as a chassis for in-situ protein synthesis.

While this study presents encouraging findings, it is important to acknowledge current limitations and areas that require further investigation. First, although in this study *C. reinhardtii* was able to maintain viability and metabolic activity under mammalian-like conditions, previous studies have shown that elevated temperatures can negatively impact recombinant protein yields. Specifically, protein production driven by nuclear expression systems was reduced at 35 °C, despite preserved culture fitness [75], and similar effects were observed in chloroplast-based expression systems at 37 °C [76]. Since the envisioned biomedical applications involve localized delivery at physiological temperatures (~ 37 °C), this represents a critical parameter that must be addressed through strain engineering optimization.

Additionally, long-term expression of recombinant genes in *C. reinhardtii* may be hindered by transgene silencing or genetic instability, leading to a progressive reduction in protein production over time. A recent proteomic study revealed substantial molecular changes and decreased transgene expression in genetically modified strains, highlighting the importance of developing more stable genetic constructs [77]. Other studies have similarly reported challenges related to transgene silencing, inefficient secretion, and low protein yields in both chloroplast and nuclear expression systems [76, 78]. The inclusion of an empty vector control can help to differentiate the effects of the genomic modification or vector components from those associated directly with the transgene expression [79, 80]. They are useful for analyzing gene silencing mechanisms and assessing potential fitness variations under different environmental conditions. The incorporation of such controls is recommended for future experimental designs to further improve the reliability of comparative analyses.

These limitations underline the need for continuous optimization of expression platforms, including the use of stabilized promoters, improved secretion signals, and selection of robust host strains to enhance protein yield and ensure long-term stability. Despite these challenges, several studies have demonstrated stable nuclear expression of recombinant proteins, including fluorescent reporters [51], VEGF [81], human IFN-α [82], human hEGF [33], α-Klotho [83], and SARS-CoV-2 spike protein [84].

Most of the application of recombinant proteins produced in *C. reinhardtii* in animal models is performed after purification of protein from cell lysates [82, 85], or application of freeze-dried protein-expressing biomass [86, 87]. Examples of directly administering *C. reinhardtii* live cells as means of recombinant protein delivery are scarce [81, 88, 89], underscoring the importance of our findings to pave the way for using mammal models to assess the safety and efficacy of this novel therapeutic approach.

For potential use in biomedical applications, *C. reinhardtii* cells might be contained within a device or compartmentalized to control and circumscribe its therapeutic effect. For this study, three previously described biomaterial-based devices were fabricated to serve as support for the recombinant microalgae: surgical sutures, alginate-based wound dressing and collagen-based dermal scaffold. The reduced thickness of the sutures limited the load of microalgae, allowing mVenus fluorescence detection only by microscopy. In contrast, the wound dressings and dermal scaffolds enabled the protein release assays, with daily replacement of DMEM-2.5% FBS, indicating that the detected mVenus fluorescence originated from *de novo* protein production within the devices. Unexpectedly, mVenus release was detected in collagen scaffolds seeded with the IN strain, suggesting recombinant protein release from damaged or lysed cells at both 30 °C and 37 °C. In contrast, apparently, the alginate matrix can effectively confine and encapsulate cells, reducing thermal stress and cell lysis. However, in both cases, chlorophyll could not be detected in the centrifuged supernatant by measuring absorbance at 680 nm (data not shown). This could be partially explained by its insolubility in aqueous media [90], which might result in its retention in the pellet composed of membranes and cellular debris.

The observation of sustained protein release up to four days from biomaterials aligns well with applications such as wound dressings for superficial wounds, where dressings are typically changed every few days. However, for other biomedical uses, including longer-term applications, further exploration is needed to extend the therapeutic window of the cells. This could involve genetic modifications or acclimation strategies to enhance tolerance to non-native conditions. For instance, different photosynthetic microorganisms have been engineered to tolerate varying salinity and exposure to non-optimal temperatures for biotechnological applications [91, 92]. Optimizing metabolic pathways to prolong cellular viability and protein synthesis in non-native conditions [93, 94] would be a crucial step toward the practical implementation of this technology for biomedical applications. It is also important to consider exploring other species of photosynthetic microorganisms with better resistance to adverse conditions [95]. For example, the cyanobacterium *Synechococcus elongatus* thrives at mammalian physiological temperatures [96] and is amenable to genetic modifications [97, 98]. Additionally, while the devices demonstrated sustained protein release in vitro, their performance in vivo remains untested. The physiological complexity of living organisms, including immune responses, metabolic interactions, and potential clearance mechanisms, could significantly influence the efficacy of these systems. Therefore, further studies of safety and efficacy using relevant preclinical animal models are necessary to validate these findings and assess the feasibility of long-term applications in clinical settings.

Although this study demonstrated recombinant protein production and release over a period of four days, its quantification remains to be elucidated. As this is critical to determine the potential therapeutic efficacy of this approach, future research should focus on obtaining quantitative data for relevant therapeutic proteins. Overall, this work provide evidence that successful genetic modification of *C. reinhardtii* allows for the synthesis and export of recombinant proteins in mammalian-like physiological conditions, supporting the idea of implanting engineered photosynthetic cells for the local and controlled release of oxygen and recombinant bioactive molecules for therapeutic purposes.

## Conclusions

The results obtained in this work show that *C. reinhardtii* may represent an excellent candidate organism for the in-situ delivery of oxygen and recombinant molecules for biomedical applications. Future approaches should focus on expanding the range of potential therapeutic proteins studying the safety and efficacy of this approach in preclinical models. Moreover, the use of inducible or environment-sensitive expression systems should be explored to enable controlled protein release in response to environmental physiological signals. By addressing these aspects, *C. reinhardtii* could serve as a novel implantable delivery system of bioactive molecules for the treatment of several pathological conditions.

## Data Availability

All data generated and analysed during this study are included in this published article and is available in the Google Drive repository, with access granted upon request by email.
